# A Roadmap toward Achieving Sustainable Environment: Evaluating the Impact of Technological Innovation and Globalization on Load Capacity Factor

**DOI:** 10.3390/ijerph19063288

**Published:** 2022-03-10

**Authors:** Abraham Ayobamiji Awosusi, Kaan Kutlay, Mehmet Altuntaş, Bakhtiyor Khodjiev, Ephraim Bonah Agyekum, Mokhtar Shouran, Mohamed Elgbaily, Salah Kamel

**Affiliations:** 1 Department of Economics, Faculty of Economics and Administrative Science, Near East University, North Cyprus, Mersin 99040, Turkey; awosusiayobamiji@gmail.com; 2 Vocational School of Health Service, European University of Lefke, Northern Cyprus, Mersin 99770, Turkey; kkutlay@eul.edu.tr; 3 Department of Economics, Faculty of Economics, Administrative and Social Sciences, Nisantasi University, Istanbul 34000, Turkey; mehmet.altuntas@nisantasi.edu.tr; 4 Department of Fundamental Economics, Tashkent State University of Economics, Tashkent 100009, Uzbekistan; b.abdullaev@akfauniversity.org; 5 Department of Nuclear and Renewable Energy, Ural Federal University Named after the First President of Russia Boris Yeltsin, 19 Mira Street, 620002 Ekaterinburg, Russia; 6 Wolfson Centre for Magnetics, School of Engineering, Cardiff University, Cardiff CF24 3AA, UK; shouranma@cardiff.ac.uk; 7 Department of Electrical Engineering, Faculty of Energy Engineering, Aswan University, Aswan 81528, Egypt; skamel@aswu.edu.eg

**Keywords:** load capacity factor, technological innovation, economic growth, nonrenewable energy usage and globalization

## Abstract

Technological innovations have been a matter of contention, and their environmental consequences remain unresolved. Moreover, studies have extensively evaluated environmental challenges using metrics such as nitrogen oxide emissions, sulfur dioxide, carbon emissions, and ecological footprint. The environment has the supply and demand aspect, which is not a component of any of these indicators. By measuring biocapacity and ecological footprint, the load capacity factor follows a certain ecological threshold, allowing for a thorough study on environmental deterioration. With the reduction in load capacity factor, the environmental deterioration increases. In the context of the environment, the interaction between technological innovation and load capacity covers the demand and supply side of the environment. In light of this, employing the dataset ranging from 1980 to 2017 for the case of South Africa, the bound cointegration test in conjunction with the critical value of Kripfganz and Schneider showed cointegration in the model. The study also employed the ARDL, whose outcome revealed that nonrenewable energy usage and economic growth contribute to environmental deterioration, whereas technological innovation and globalization improve the quality of the environment. This study validated the hypothesis of the environmental Kuznets curve for South Africa, as the short-term coefficient value was lower than the long-term elasticity. Furthermore, using the frequency-domain causality test revealed that globalization and economic growth predict load capacity in the long term, and nonrenewable energy predicts load capacity factors in the long and medium term. In addition, technological innovation predicts load capacity factors in the short and long term. Based on the findings, we propose that policymakers should focus their efforts on increasing funding for the research and development of green technologies.

## 1. Introduction

Paradigm transitions and structural gradual transformations toward the services and information sector from the intensive-pollution sector can help to minimize aggregate negative externalities to all economies around the world. Globalization, the advancement in technology, and the implementation of environmental legislation have greatly decreased energy-related greenhouse gas emissions in both industrialized and emerging nations (e.g., South Africa). Moreover, the composition of total waste has changed from greenhouse gas (GHGs) emissions toward other forms of pollutants such as effluents and solid waste [1,2,3]. These conclusions suggest that the overall environmental pollution remains significantly high, necessitating more reform initiatives under the defined sustainable development goals.

According to Chen et al. [4], the best way to achieve sustainable growth and decent work (SDG-8) is to increase transparency in the financial framework using technological processes. Furthermore, industrial actors can lower their pollution by promoting the use of advanced technologies that support clean and affordable energy (SDG-7). In addition, Sinha et al. [5] proposed that nations that have achieved the SGD-7 should be motivated to pursue a sustainable environment (SGD-13). Thus, the transition from fossil-based energy to energy-efficient technology will be realized through innovations that decrease environmental deterioration, create green employment, and improve environmental quality [6]. Hence, there are several determinants of environmental degradation, some of which are the level of income, nonrenewable and renewable energy, globalization, urbanization, and technological innovation.

**Figure 1:**
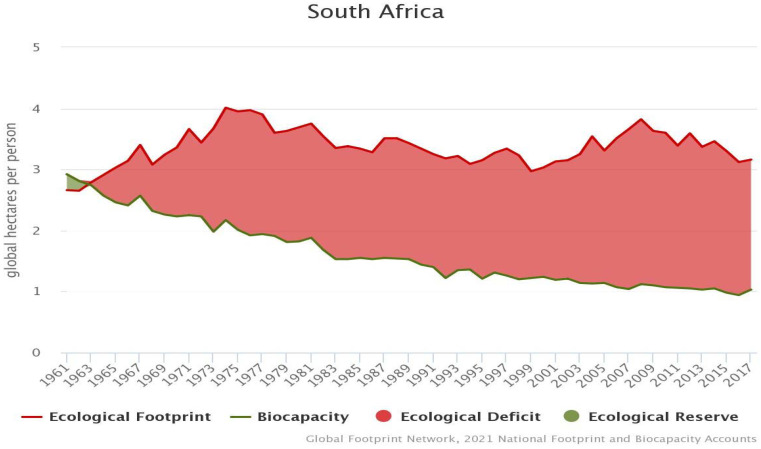
The trends in ecological footprint and biocapacity in South Africa.

Presently, South Africa is an emerging economy with a higher level of income when compared to other economies in the sub-Saharan Africa region. However, this growth is achieved by the consumption of fossil fuels, which contributes about 95.29% of the country’s energy mix (World data, 2021). This contributes to the increase in ecological footprint and reduction in biocapacity in South Africa, as illustrated in Figure 1. The increasing level of ecological deficit compelled the authors to perform empirical research for South Africa by investigating the effect of globalization, nonrenewable energy, technological innovation, and economic expansion on environmental degradation.

Much research has examined the effect of several determinants on carbon emissions [3,7,8,9]. Carbon emission only accounts for a significant proportion of GHGs, which is insufficient to explain and appraise the total environmental deterioration [10]. However, several researchers [11,12] have argued that ecological footprint is a more comprehensive measure for environmental deterioration. However, the account for ecological footprint involves two measurements: ecological footprints and biocapacity. This measurement covers different aspects of the ecosystem: ecological footprints cover the demand side of nature, whereas biocapacity covers the supply side. Many studies have examined the effect of several determinants on ecological footprint [10,12,13,14] but have neglected the supply side (biocapacity). As a result, there is a need to develop a more suitable and reliable evaluation for assessing environmental quality. In light of this, the load capacity factor was suggested by [15]. Fareed et al. [16] argued that the load capacity factor reflects a country’s capacity to keep its population in conformity with their modern lifestyles. The load capacity factor is computed by dividing the supply side (biocapacity) from the demand side (ecological footprint) [17]. The state of the ecosystem is unsustainable when the load capacity is less than 1 and it is said to be sustainable when the load capacity is more than 1. As a result, the sustainability threshold equals one. As a premise of the aforementioned argument, the load capacity factor is a more comprehensive assessment than carbon emissions and ecological footprint. Hence, in comparison to previous research, we conduct a more complete and broad investigation.

Regarding the above explanation, the goal of this present study is to investigate the effect of nonrenewable energy, globalization, economic growth, and technological innovation on load capacity factors using the ARDL method for a dataset spanning from 1980 to 2017. This present study’s significant contribution toward the corpus of energy and environmental literature is as follows: (i) No attempt is made toward examining the role of technological innovation on load capacity for any emerging economy (e.g., South Africa). Load capacity is used as the metric for environmental degradation. (ii) No study has been undertaken with regard to investigating the role of globalization on environmental degradation, using load capacity factor as the metric for environmental degradation. (iii) This current study scrutinizes whether the EKC hypothesis is valid for load capacity factors within the context of Narayan and Narayan’s [18] approach. As a result, the research addresses possible multicollinearity issues. (iv) Finally, using the frequency domain causality, this study attempts to uncover the causality association between load capacity factor and its regressors. However, the novelty of the approach is that it uncovers the causality interaction at different frequencies (short, medium, and long run), which cannot be detected by conventional causality tests.

The remaining sections of this study are compiled as follows: Section 2 details a synopsis of related studies. The data and methods are presented in Section 3, and the theoretical underpinning is discussed. Section 4 portrays the findings and discussion, and the conclusion is discussed in Section 5 of this study.

## 2. Literature Review

In the section of the research, we review the literature of prior studies investigating the subject matter, which is classified into three sections: economic–energy–environmental degradation nexus, technological innovation–environmental degradation nexus, and globalization–environmental degradation nexus.

### 2.1. Economic–Energy–Environmental Degradation Nexus

A growing body of research has attempted to look at the basic interconnections between economic–energy–environmental degradation. Oladipupo et al. [8] contended that economic growth and nonrenewable energy degrade the environment in Japan for the period between 1965 and 2019. Bekun et al. [7] studied coal energy consumption and economic growth as a determinant of carbon emission for South Africa over the period between 1980 and 2017. They concluded that coal energy consumption and economic growth increase carbon emissions. Likewise, Ramzan et al., [18] scrutinized the interconnection of energy usage and carbon emission in Latin American economies covering the period between 1980 and 2017. They claimed that energy consumption and economic growth stimulate carbon emissions. In [10], the role of energy usage and economic growth was considered on Brazil’s ecological footprint. They established that energy usage and economic expansion worsened the quality of the environment for the period between 1983 and 2017. Akadiri and Adebayo [19] studied the nexus of nonrenewable energy–economic growth–carbon emission in India. Using the Nonlinear Autoregressive Distributed Lag (NARDL) to investigate the period ranging from 1970 to 2018, these authors also confirmed that nonrenewable energy and economic growth stimulate carbon emissions.

Ayobamiji and Kalmaz [3] studied the role of energy consumption and economic growth association on carbon emission for Nigeria. Deploying the wavelets approach, these authors argued that economic growth and energy consumption encourage carbon emission in Nigeria. In another study, the dynamics between economic growth, fossil fuel, and ecological footprint were studied by [20]. Their outcomes also confirmed that economic growth and fuel contribute to the increase in ecological footprint in China. Kihombo et al. [14] scrutinized the linkage between economic growth, energy consumption, and ecological footprint in West Asian and the Middle East (WAME) economies over the period between 1990 and 2017. Their outcomes concluded that energy consumption increases the ecological footprint. Economic growth also contributes to the increase in ecological footprint.

### 2.2. Technological Innovation–Environmental Degradation Nexus

Many investigations have been directed toward considering the underlying linkages between technological innovation and environmental quality. Kihombo et al. [11] studied the connection between technological innovation and ecological footprint from 1990 to 2017. They confirmed that technological innovation reduces the ecological footprint in WAME economies. Yang et al. [21] scrutinized the effect of technological innovation on Brazil, India, China, and South Africa’s ecological footprint over the period between 1990 and 2016. They argued that technological innovation reduces the ecological footprint. In addition, [13] tested the effect of technological innovation on Pakistan’s ecological footprint over the period between 1992 and 2018 and established that technological innovation contributes to the ecological footprint. Destek and Manga [22] studied the effect of technological innovation on ecological footprint and carbon emission in large emerging markets over the period between 1995 and 2016. They concluded that technological innovation reduces carbon emissions, whereas technological innovation does not impact the ecological footprint.

Ahmad et al. [23] investigated the involvement of technological innovation in achieving a sustainable environment in twenty-two economies from 1984 to 2016. Their outcome elucidated that technological innovation decreases the ecological footprint. Conversely, ref. [24] studied the role of technological innovation on the Asia Pacific Economic Cooperation’s ecological footprint. The empirical outcome resolved that technological innovation increases the ecological footprint. Wahab et al. [25] established that technological innovation reduced consumption-based carbon emissions in G-7 economies for the timeframe between 1996 and 2017. However, the research of [26] concluded that technological innovation increased carbon emissions in Brazil for the period between 1990 and 2018. Likewise, [27] confirmed that technological innovation increases carbon emissions in Japan.

### 2.3. Globalization–Environmental Degradation Nexus

Several research studies have been carried out to scrutinize the underlying linkages between globalization and environmental degradation. Yuping et al. [9] examined the effect of globalization on Argentina’s carbon emissions. They concluded that globalization reduces carbon emissions. Likewise, [28] suggested a similar outcome in Argentina for the period between 1980 and 2017. Conversely, ref. [12] studied the role of globalization on Turkey’s ecological footprint. The empirical outcome confirmed that globalization increases the ecological footprint. Likewise, [29] confirmed that globalization increases carbon emissions in Australia. Coelho et al. [30] investigated the involvement of globalization in achieving a sustainable environment in South Korea from 1980 to 2018. Their outcome elucidated that globalization increases the ecological footprint. Adebayo et al. [31] established that globalization does not affect carbon emissions in South Africa. Likewise, the study of [32] in Malaysia confirmed that globalization does not affect the ecological footprint.

Ansari et al. [33] studied the role of globalization on ecological footprint. Using the DOLS and FMOLS approach, these authors argued that globalization reduces the ecological footprint in twenty-two selected economies. In another research, the interaction between globalization and ecological footprint was scrutinized by [34]. Their outcomes also confirmed that globalization decreases the ecological footprint in twenty-three emerging economies.

We observed that the findings obtained from the reviewed literature provides mixed outcomes due to the period of study, country of study, econometric approach, and indicator for measuring environmental degradation. Meanwhile, the indicators for environmental pollution such as ecological footprint and carbon emissions have been the subject of recent research. Technological innovation and globalization, on the other hand, can have severe consequences on the sea, forests, and other natural resources. To put it another way, the influence of technological innovation and globalization on the capacity of the natural resource supply (biocapacity) must be considered. This current study employed the load capacity factor, which includes both the biocapacity and the ecological footprint, to address this gap in the literature. We hope to introduce a new dimension to the globalization–environment connection as well as the technological innovation–environment nexus, which contributes to the current literature by looking at the influence of technological innovation and globalization on load capacity factor.

## 3. Theoretical Framework, Model Construction, and Model

### 3.1. Empirical Framework

The South African economy has experienced a continuous increase in its economy. However, this growth is fueled by the consumption of nonrenewable energy, notably coal, which contributes about 70% of the country’s energy mix [7]. The side-effect of this growth has led to the surge in degradation of the environment. In curbing this side-effect, there is a need for the energy mix of the county to be changed. However, for these changes to be achieved, there is a need for the industrial and manufacturing sectors to reduce the use of nonrenewable energy by encouraging the use of recently advanced and innovative green energy solutions. Meanwhile, to tackle the problems of environmental degradation, the country needs to make efforts toward ensuring that the technological innovation of the country continues to evolve; however, this effort will also help to increase the growth pattern of the economy. This effort comes in the form of integrating the economy with the rest of the world. As South Africa is an emerging economy, its integration with the rest of the world will improve the technological advancement of this nation and also help address the environmental concern of the country. Based on the theoretical perspective of the study, we tried to investigate the EKC hypothesis using Narayan and Narayan’s [35] approach. There is a need to present this interaction mathematically, which is showcased in the model as follows:

(1) $$LOCAP_{t}=f\left(GDP_{t},NREN_{t},TEC_{t},\;GLO_{t}\right)$$

(2) $$LOCAP_{t}=\vartheta_{0}+\vartheta_{1}GDP_{t}+\vartheta_{2}NREN_{t}+\vartheta_{3}TEC_{t}+\vartheta_{4}GLO_{t}+\varepsilon_{t}\;$$

where subscript *t* and *ε* depict the study period (1980–2017) and error term, respectively; the coefficients of the parameters are indicated as $\vartheta_{1,..,4}$. GDP, NREN, TEC, GLO, and LOCAP indicate economic growth, nonrenewable energy, technological innovation, globalization, and load capacity factor, respectively. However, this study expects that the sign of $\vartheta_{1}$ is negative, in which GDP contributes to environmental degradation, i.e., $\;\vartheta_{1}=\frac{\partial LOCAP}{\partial GDP}<0$. In addition, the anticipated sign of *ϑ*_2_ is negative, i.e., $\;\vartheta_{2}=\frac{\partial LOCAP}{\partial NREN}<0$. The expected direction for *ϑ*_3_ could be a beneficial or adverse effect, i.e., $\vartheta_{3}=0<\frac{\partial LOCAP}{\partial TEC}>0$. For instance, the development of renewable energy through technological innovations allows the use of clean energy options for production operation and results in increasing the quality of the environment. Meanwhile, if the innovations are focused on the development of nonrenewable energy, the level of industrial pollution will increase. Finally, the predicted sign for $\vartheta_{4}$ could be positive or negative, i.e., $\vartheta_{4}=0<\frac{\partial LOCAP}{\partial GLO}>0$. For instance, if the globalization policies are strict toward the dirty industries, the level of environmental degradation will reduce, whereas, if it is lax, the environmental degradation will increase.

### 3.2. Data Description

The effect of economic growth, nonrenewable energy, technological innovation, and globalization was investigated on the load capacity factor in South Africa for the period between 1980 and 2017. Owing to the unavailable data for globalization and technological innovation, the study period for this research began in 1980, and the study could not stretch beyond 2017, due to unavailable data for load capacity factor. The data were sourced from the World Bank database for economic growth and technological innovation. Meanwhile, nonrenewable energy, globalization, and load capacity factors were sourced from the BP database, KOF index, and Global Footprint Network, respectively. To reduce the possibility of aberrations during the estimation, the parameters were transformed into their natural logarithms. Table 1 showcases the description of the variables used.

### 3.3. Methods

Before utilizing the bounds testing procedure, to identify the order of integration of the parameters under examination, the stationary assessment must be performed on them. To check the asymptotic nature and integration order of all parameters under investigation, this study used Phillips–Perron (PP) and Kwiatkowski–Phillips–Schmidt–Shin (KPSS) unit root tests. These tests lack the ability to detect structural break, which could produce erroneous regressions. For this purpose, this study employed the Zivot Andrew (ZA) unit root test, which can uncover a structural break during regression, thereby facilitating the elimination of erroneous regressions.

Furthermore, this bound testing procedure was used in this research to look at the relationship between the variables under consideration in the long term. Based on Pesaran et al. [36], the ARDL bound testing technique was specified in this regard.

(3) $$\;\;LOCAP_{t}=\theta_{0}+\overset{p}{\underset{l=1}{\sum }}\theta_{1}\Delta LOCAP_{t-1}+\overset{p}{\underset{i=1}{\sum }}\theta_{2}\Delta GDP_{t-1}+\overset{p}{\underset{i=1}{\sum }}\theta_{3}NREN_{t-1}+\overset{p}{\underset{i=1}{\sum }}\theta_{4}\Delta TEC_{t-1}+\overset{p}{\underset{i=1}{\sum }}\theta_{5}\Delta GLO_{t-1}\;+\pi_{1}LOCAP_{t-1}+\pi_{2}GDP_{t-1}+\;\pi_{3}NREN_{t-1}+\pi_{4}TEC_{t-1}+\pi_{5}GLO_{t-1}+\epsilon_{t}$$

where Δ and i indicate first difference and lag length, respectively. $\theta_{1,..,4}$ and $\pi_{1,..,4}$ indicate the short and long-term coefficients, respectively. Meanwhile, the null hypothesis for the long-term association is $\theta_{1}$ = $\theta_{2}$ = $\theta_{3}$ = $\theta_{4}$ = 0 against the alternate hypothesis ($\theta_{1}$ ≠ $\theta_{2}$ ≠ $\theta_{3}$ ≠ $\theta_{4}$ ≠ 0). Into the short-term parameter of the ARDL, the ECM (error correction model) is integrated, thereby converting Equation (3) to Equation (4) as:

(4) $$\begin{array}{ll} LOCAP_{t}=\theta_{0}+ & \overset{p}{\underset{l=1}{\sum }}\theta_{1}\Delta LOCAP_{t-1}+\overset{p}{\underset{i=1}{\sum }}\theta_{2}\Delta GDP_{t-1}+\overset{p}{\underset{i=1}{\sum }}\theta_{3}NREN_{t-1}+\overset{p}{\underset{i=1}{\sum }}\theta_{4}\Delta TEC_{t-1}+\overset{p}{\underset{i=1}{\sum }}\theta_{5}\Delta GLO_{t-1}\\ & \;+\pi_{1}LOCAP_{t-1}+\pi_{2}GDP_{t-1}+\;\pi_{3}NREN_{t-1}+\pi_{4}TEC_{t-1}+\pi_{5}GLO_{t-1}+\pi_{6}ECT_{t-1}\\ & \;+\epsilon_{t} \end{array}$$

where ECT indicates the error correction term, and its coefficient is represented as $\pi_{6}$. However, the value of the estimated F and T statistics determines whether the null hypothesis is rejected or accepted. Refuting the null hypothesis, the estimated F and T statistics will have to be superior to the upper critical bound level. Cointegration, on the other hand, does not occur whenever the estimated statistics of T and F are smaller than that of the lower bound. Furthermore, when the estimated F and T statistics value falls between the lower and higher bounds, the bound test seems to be inconclusive.

Finally, we employed the frequency domain causality test to determine the causality interaction between load capacity and the other parameters. The novelty of the approach is that it can uncover causal interaction at different frequencies (long, medium, and short term), which cannot be detected by the conventional causality tests. The approach is centered on the reconstructed Vector Autoregressive (VAR) interaction between *x* and *y*, which is written as:

(5) $$x_{t}=\theta_{1}x_{t-1}+.\ldots +\theta_{1}x_{t-1}+\beta_{1}y_{t-1}+\ldots +\beta_{l}y_{t-1}+\varepsilon_{t}$$

To select the optimal lag (l), the study used the Akaike Information Criterion (AIC). The null hypothesis is stated as:

(6) $$H_{0}:R\left(\omega \right)\beta =0$$

The vector that connects the *y* coefficients is denoted as  β:

(7) $$R\left(\omega \right)=\frac{\cos\left(\omega \right)\cos\left(2\omega \right)\ldots .\cos\left(l\omega \right)}{\sin\left(\omega \right)\sin\left(2\omega \right)\ldots .\sin\left(l\omega \right)}$$

t =$\frac{2\pi }{\omega }$, which indicates t as period and is connected to the frequency ω.

## 4. Results and Discussion

**Figure 2:**
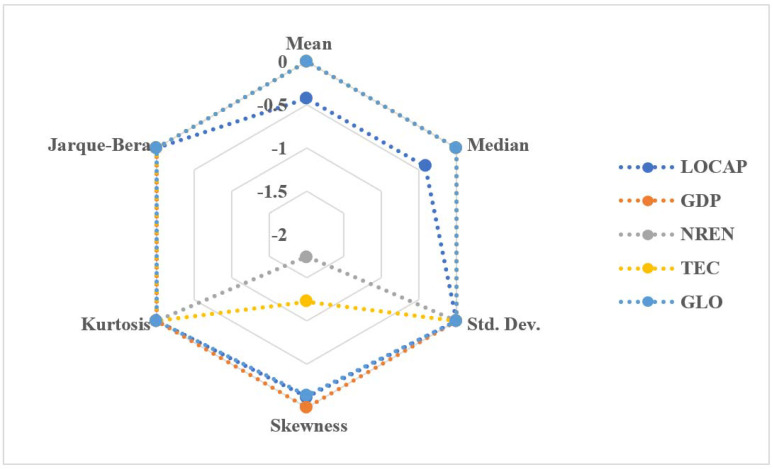
RADAR chart.

Our empirical investigation for South Africa started with examining the descriptive analysis that provides information about the nature of the variables under consideration, as shown in Table 2. It reveals that technological innovation has the highest average value of 3.854, whereas nonrenewable energy usage has the second-highest average value of 3.746. Technological innovation also ranges between 3.497 and 4.006. However, globalization’s average value is 1.709, ranging from 1.850 to 1.545. Economic growth, on the other hand, is associated with the average value of 3.692, with the minimum and maximum values of 3.622 and 3.760, respectively. Furthermore, in the case of South Africa, the average of load capacity factor is −0.425, with the value of minimum and maximum as −0.299 and −0.548, respectively. During the period 1980–2017, the nonrenewable energy usage had a minimum value of 3.380 with a maximum value of 3.869. Meanwhile, the median values of 3.761, −0.416, 1.736, 3.862, and 3.682 are nonrenewable energy usage, load capacity factor, globalization, technological innovation, and economic growth, respectively. In addition, the standard deviation of load capacity factor, globalization, economic growth, nonrenewable energy usage, and technological innovation are 0.078, 0.124, 0.046, 0.099, and 0.115, respectively. Load capacity factor, nonrenewable energy usage, technological innovation, and globalization are negatively skewed, while economic growth is positively skewed. However, for the kurtosis of the concern variables, load capacity factor, economic growth, and globalization are platykurtic in nature, whereas nonrenewable energy usage and technological innovation are leptokurtic in nature. From the skewness and kurtosis, all variables are normally distributed except for nonrenewable energy usage and technological innovation, which is backed by the Jarque–Bera test and its probability value. Moreover, as shown in Figure 2, the RADAR chart offers a graphical representation of the observed series’ descriptive statistics.

This research’s empirical analysis also necessitates the assessment of the stochastic nature of each variable by employing stationary tests. Based on this context, the three distinct stationary tests, namely KPSS, PP, and ZA unit root tests, were used in this study. In Table 3, the outcome of the KPSS and PP is reported, which indicates that all considered variables are stationary at first difference, except for nonrenewable energy usage that is stationary at level, indicating a mixed order of integration. However, these unit root tests are regarded as the conventional unit root testing procedure that could produce inaccurate estimates, which could lead to erroneous outcomes during regression. The ZA unit root test was used in this investigation for this reason, whose outcomes are reported in Table 4. It shows that at level, we reject the null hypothesis of non-stationarity for nonrenewable energy usage with the structural break in 2005. Moreover, the null hypothesis of non-stationarity was rejected at first difference with the structural break in 2009, 2009, 1999, 1993, and 2001 for load capacity factor, economic growth, nonrenewable energy usage, technological innovation, and globalization, respectively. The variables in this situation are consistent with zero means and constant variance, which makes it desirable. Next, the cointegration analysis can now be examined.

**Figure 3:**
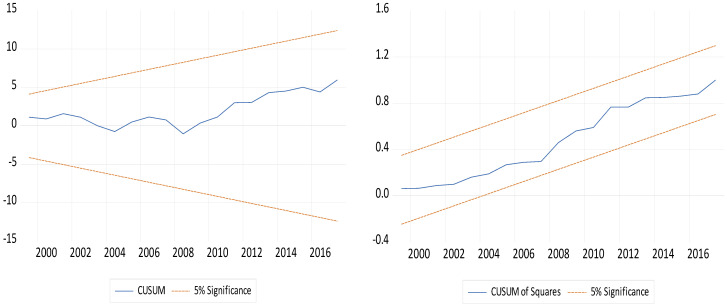
Stability test.

Table 5 explores the outcome of the bound test for South Africa. This study employed the critical values of [37] to compare the F-statistics and T-statistics. At a 1% significance level, the F-statistics of 7.947 exceeds the critical value of 5.06, which suggests that the null hypothesis of no cointegration is rejected. In addition, at a 1% level of significance, the T-statistics of −6.936 supersedes the critical value of −4.6, indicating the rejection of the null hypothesis of no cointegration. Hence, based on these outcomes, we conclude that there is a cointegrating association between load capacity factor and its regressors. Furthermore, the post-estimation test (diagnostic tests) indicates that there is no presence of serial correlation, heteroskedasticity, and incorrect functional form. The residuals of the model are also normally distributed and stable, as indicated by the CUSUM and CUSUMSQ test, which is shown in Figure 3. Having established a cointegrating association, the next analysis is to determine the effect of these regressors on load capacity factors.

Table 6 summarizes the outcome of the ARDL estimators. As seen in Table 6, in the long run, economic growth and nonrenewable energy decrease load capacity factor. Meanwhile, technological innovation and globalization increase load capacity factor. In addition, as expected, the error correction term reveals a negative and statistically significant having its value has 0.572 (57.2%), demonstrating the imbalance that may occur in the short period, where the convergence process would require about a year and a half. As a result, the process of convergence is quite average, and the regressors impact load capacity factor with a year and a half of lag.

For a more robust discussion, a negative association is evident between economic growth and load capacity factor both in the short term and long term. The load capacity factor will decrease by 1.857%, as a result of an increase in economic expansion by 1% in the short term, whereas in the long term, as a result of the increase in economic expansion by 1%, there will be a reduction of 1.592% in load capacity factor. Therefore, continuous economic activity contributes to environmental degradation in South Africa both in the short run as well as the long term. When comparing the short and long-run effects, the short-run negative effect supersedes that of the long run. Under this scenario, it shows that environmental degradation is reducing over time, confirming the validity of the EKC hypothesis. Hence, from the outcome of the estimator, we conclude that the EKC hypothesis is valid in South Africa. The outcome of this study is consistent with the outcomes of Usman et al. [38] and Rafindadi and Usman [39] for carbon emissions, but not with those of Rjoub et al. [40] in Sweden. Moreover, the possible reasons for the inconsistency in results could be the use of different techniques, the combination of variables used during the study period, and many more. Regardless of the validity of EKC, income promotes environmental deterioration in both the short and long run, indicating the scale effect. This indicates that the South African government is pursuing a pro-growth policy. Furthermore, the economic growth achieved in South Africa is at the expense of environmental challenges such as pollution on land, sea, and air.

South Africa, as an emerging nation, utilizes a large number of natural resources and depends on energy resources, which are carbon-intensive, to increase its economy. South Africa’s rapid expansion has been concentrated on resource-intensive industries, and exports have practically surpassed their limits, leading to environmental challenges. Thus, South Africa’s economic boom, notably throughout the 2000s, has exacerbated environmental deterioration. This suggests that the growing per capita income does not inevitably result in a more sustainable environment. Thus, there is a need for the South African government to adopt energy-related environmental policies.

As expected, the impact of nonrenewable energy usage on load capacity factor is negative both in the short and long run, as reported in Table 6. Precisely, the load capacity factor will decrease by 0.187%, as a result of an increase in nonrenewable energy by 1% in the short and long run. Based on this finding, it is suggested that the usage of nonrenewable energy is the primary cause of environmental deterioration in South Africa. The conclusions of this study are compatible with prior literature, which states that increasing the usage of nonrenewable energy causes a deterioration in the environment [7,31,35,41,42]. Regarding the negative impact of nonrenewable energy usage on load capacity factor, one possible rationale could be that the country is tranquil in its overdependence on fossil fuel. For instance, nonrenewable energy consists of 95.29% of the energy mix in South Africa, which is 70.81% (coal), 21.44% (oil), and 3.04% (natural gas) in 2017. To achieve a sustainable environment and development, the South African government needs to reduce its dependence on nonrenewable energy usage.

Moreover, the coefficient of globalization is significant and positive on load capacity factor both in the short and long run. To be precise, the increase in the level of globalization (economic, political, or social component) by 1% will increase load capacity by 1.481% in the long term and 1.481% in the short term, indicating that globalization in South Africa has reached a level where it can contribute to the quality of the environment. Hence, globalization contributes to the reduction in environmental deterioration in South Africa. Considering that both the level of degradation in the environment and globalization index continues to increase over time, this current study contradicts the pollution-haven hypothesis, which concludes that globalization opens an opportunity for foreign dirty industries to increase their activities in emerging economies, such as South Africa, and contributes to pollution, whereas it supports the pollution-halo hypothesis, which emphasizes that globalization significantly contributes to the quality of the environment. This hypothesis argues that foreign direct investment (FDI) from multinational corporations allows the transfer of greener technologies to the host country. The transfer of technologies consists of green technologies such as pollution reduction technologies and renewable energy technologies, as well as improved energy efficiency technologies, that reduce the need for conventional sources of energy. This argument serves as the possible reason for the positive role of globalization toward load capacity factor in South Africa. South Africa can potentially improve its pollution-reduction possibilities by using the benefits of its integration with the BRICS economies. The establishment of the BRICS gives these nations the chance to discuss their energy policies and work together to enhance their energy prospects. In addition, the study coincides with the research of Güngör et al. [43] for ecological footprint and Salahuddin et al. [44] for carbon emissions, which established a negative connection between environmental degradation and globalization in South Africa; however, the study of Adebayo et al. [31] established an insignificant association between carbon emissions and globalization in South Africa.

Moreover, the coefficient of technological innovation is negative and significant on load capacity factor in the short term, whereas, in the long term, the coefficient of technological innovation is significant and positive on load capacity factor. To be precise, the increase in technological innovation by 1% will decrease load capacity by 0.270% in the short run, and in the long run, load capacity increases by 0.169. This outcome revealed that technological innovation contributes to the detrimental effect on the environment in the short term; meanwhile, in the long term, it contributes to the quality of the environment. Thus, the short degradation of technological innovation has been addressed in the long term. This outcome makes sense because, as an emerging economy, South Africa aims to experience growth, so major technological innovations are channeled toward dirty industries, leading to environmental concerns (i.e., pro-growth agenda). However, the rise in the level of innovation over time will lead to the improvement in better technologies that require fewer resources for production, which will subsequently reduce the degradation of the environment. In addition, this improvement will encourage the usage of green technologies, which will reduce the usage of polluting energy sources. This outcome is consistent with the research of [11,21].

**Figure 4:**
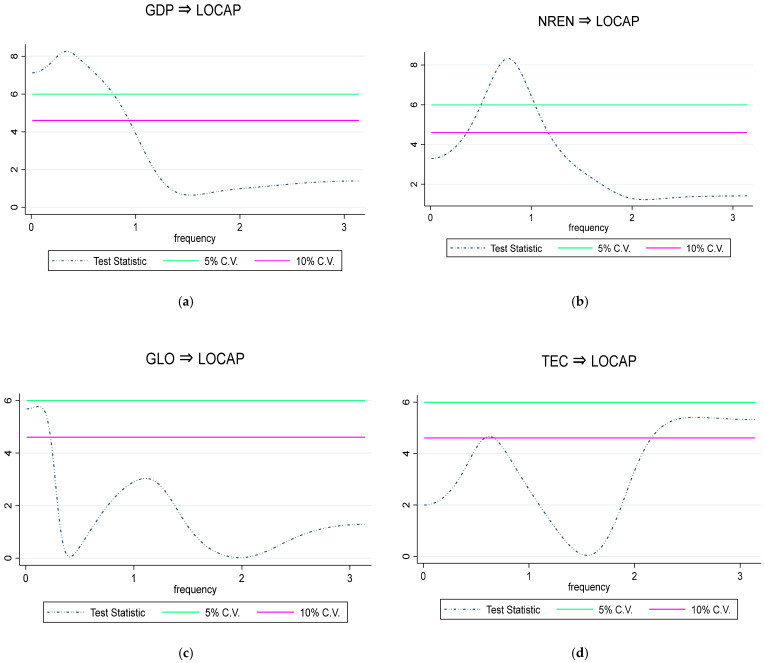
(**a**) Causal interaction of economic growth and load capacity factor. (**b**) Causal interaction of nonrenewable energy and load capacity factor. (**c**) Causal interaction of globalization and load capacity factor. (**d**) Causal interaction of technological innovation and load capacity factor.

As the long-run impact of the regressors on load capacity factor has been uncovered, this study also investigated the causal effect of these regressors on load capacity in the long, medium, and short run using the frequency-domain causality test. This outcome of the test is presented in Figure 4a–d. The lime and pink solid line signify the 5% and 10% level of significance, respectively, whereas the T-statistics of the Breitung and Candelon [45] frequency-domain causality test is denoted as blue curved dotted line. As seen in Figure 4a, which presents the outcome of the causal association from economic growth to load capacity, it indicates that the hypothesis of noncausality relationship from economic growth to load capacity is rejected in the long run. This indicates that the economic growth predicts load capacity factor only in the long term in South Africa. Meanwhile, in the long and medium run, as reported in Figure 4b, non-renewable energy usage Granger causes load capacity factor. This reveals that nonrenewable energy usage can forecast changes in load capacity factors in the long and medium run. In addition, Figure 4c shows the evidence of a causal relationship from globalization to load capacity factor in the long run. It indicates that globalization is a predictor of load capacity factors in the long run. Finally, as expected, both in the short and long run, it is evident that there is a causal relationship from technological innovation to load capacity factor, as uncovered in Figure 4d. It shows that technological innovation can forecast major variations in load capacity factors in the short and long run. 

## 5. Conclusions

Recent research has emphasized the necessity to increase technological innovation to decrease environmental damage. However, empirical research on the relationship between technological innovation and CO_2_ emissions has produced inconsistent results. Likewise, empirical research on the interaction between ecological footprint and technological innovation has uncovered mixed outcomes. However, no empirical studies have examined the interaction between technological innovation and load capacity. In light of this, employing the dataset ranging from 1980 to 2017, this study investigated the impact of economic growth, technological innovation, nonrenewable energy usage, and globalization on the load capacity factor in South Africa. To do so, this study employed the KPSS, PP, and ZA unit root tests to determine the integration order of economic growth, technological innovation, nonrenewable energy usage, globalization, and load capacity factor. In addition, evidence from the bound cointegration test in conjunction with Kripfganz and Schneider [42] showed cointegration in the model. Next, the ARDL estimator was employed, which generated coefficients that showed that technological advancement and globalization could assist in fulfilling the aspiration of a sustainable environment by increasing the load capacity factor in South Africa. Nonrenewable energy usage and economic growth, on the other hand, help to raise environmental degradation as they decrease the load capacity factor in South Africa. Furthermore, the outcome suggests the presence of EKC in South Africa. Finally, the outcome of the Breitung and Candelon [41] frequency-domain causality test revealed that globalization and economic growth can predict load capacity in the long run, while nonrenewable energy can predict load capacity factors in the long and medium run. In addition, technological innovation can forecast major changes in load capacity factors in the short and long run. 

### Policy Directions

First, having established the validity of EKC in South Africa, it does not suggest that environmental issues in South Africa would be solved seamlessly. Neglecting environmental issues in South Africa for the sake of economic growth could potentially contribute to even more significant issues in the coming years. With the short- and long-term negative effects of GDP on the sustainable environment, South African authorities should adhere to environmental laws and regulations by formulating guidelines in the areas of natural resource management, education, and energy. In addition, while adopting economic growth initiatives that adversely impact ecological sustainability, South African authorities should take caution.

Second, the adverse effect of nonrenewable energy on the quality of the environment shows that nonrenewable energy is unsustainable. South Africa needs to reduce their reliance on nonrenewable energy to fulfill the nation’s energy demands. There is a need for the authorities of South Africa to be committed to increasing the country’s investment in renewable energy, by also enacting and executing supportive policies with the sole purpose of overcoming the conventional obstacles that have hampered the development and adoption of renewable energy in South Africa.

Third, as technological innovation is sustainable in South Africa, it should be promoted by increasing funding for the research and development of green technologies. There is also a need for the government of South Africa to foster the advancement of technologies that make renewable energy more accessible and cost-effective. Furthermore, authorities should encourage researchers and institutions to create energy-saving technologies, and such incentives can come in the form of tax exemptions and subsidies. A close partnership between universities and businesses, as well as the provision of research grants, could help to raise the technological innovation level.

Fourth, accelerating the rate of globalization could mitigate the environmental effects of nonrenewable energy and economic expansion through the advancement in technology connected to the process of globalization. As a result, to maximize the benefits of globalization, we recommend that carbon taxes should be fostered, energy-intensive operations should be effectively supervised, and environmental laws should be strictly enforced to avoid the negative impact of globalization on the environment as a result of the anticipated accelerated upsurge in energy utilization. 

Finally, this finding opens up new directions for an investigation into the matter. The effect of economic growth, technological innovation, globalization, and nonrenewable energy usage on load capacity factors can be investigated by other future studies by employing different methodological techniques or can be focused on by individual countries or groupings of countries. The drawback of this present study relates to the period of study. Future studies can expand the duration of study.

## Figures and Tables

**Table 1:** Description of the variable.

Indicators	Description	Unit	Sourced
LOCAP	Load capacity factor	Biocapacity divided by ecological footprint	Global Footprint Network
NREN	Nonrenewable energy	KWPH	BP database
GDP	GDP per capita	constant 2015 USD	World Bank Database indicators
TEC	Technological innovation	Added both resident and non-resident patent applications	
GLO	Globalization index	Index based on economic, political, and social	KOF Globalization Index

**Table 2:** Descriptive statistics.

	LOCAP	GDP	NREN	TEC	GLO
Mean	−0.425	3.692	3.746	3.854	1.709
Median	−0.416	3.682	3.761	3.862	1.736
Maximum	−0.299	3.760	3.869	4.006	1.850
Minimum	−0.548	3.622	3.380	3.497	1.545
Std. Dev.	0.078	0.046	0.099	0.115	0.124
Skewness	−0.119	0.138	−1.743	−1.224	−0.134
Kurtosis	1.592	1.636	6.952	5.249	1.230
Jarque–Bera	3.228	3.065	43.978	17.490	5.075
Probability	0.199	0.216	0.000	0.000	0.079
Observations	38	38	38	38	38

**Table 3:** ADF and PP tests.

Variables	KPSS		PP	
	Level	*Δ*	Level	*Δ*
LOCAP	0.073	0.431 *	−2.954	−7.974 *
GDP	0.203	0.262 *	−1.725	−4.288 *
NREN	0.254 *	0.024	−3.673 **	−5.650 *
GLO	0.097	0.726 *	−1.975	−5.029 *
TEC	0.162	0.199 **	−2.359	−4.155 *

Note: ** and * portray significance levels of 0.05 and 0.01, respectively.

**Table 4:** Structural break unit-roots outcome.

	I(0)	I(1)
LOCAP	−5.366 (2006)	−7.175 * (2009)
GDP	−3.582 (1990)	−5.980 * (2009)
NREN	−4.964 (2005) **	−5.429 ** (1999)
GLO	−3.664 (2006)	−5.925 * (1993)
TEC	−4.206 (1990)	−6.770 * (2001)

* and ** portrays significance level of 0.01 and 0.05 respectively; structural breaks are in parentheses.

**Table 5:** ARDL approach to cointegration.

F-Statistic	7.947 *					
T-Statistic	−6.936 *					
Kripfganz and Schneider Critical Values						
	1%		5%		10%	
	LB	HB	LB	HB	LB	HB
F-statistic	3.74	5.06	2.86	4.01	2.45	3.52
T-statistic	−3.43	−4.6	−2.86	−3.99	−2.57	−3.66
Diagnostic Check						
χ^2^ Normality	1.206 (0.547)					
χ^2^ LM	0.490 (0.621)					
χ^2^ Heteroscedasticity	0.716 (0.742)					
χ^2^ Ramsey	1.931 (0.176)					

* portrays significance level of 0.01.

**Table 6:** ARDL estimator outcome.

Variable	Coefficients	T-Statistics
GDP	−1.592 **	−2.839
NREN	−0.187 **	−2.407
GLO	1.481 *	3.154
TEC	0.169 ***	2.060
ΔGDP	−1.857 *	−4.005
ΔNREN	−0.187 *	−3.066
ΔGLO	1.481 *	4.488
ΔTEC	−0.270 *	−3.672
ECT(−1)	−0.572 *	−6.936

*, ** and *** portray significance levels of 0.01, 0.05 and 0.1, respectively.

## Data Availability

Data used are available in the text.
